# A population-based analysis of germline *BRCA1* and *BRCA2* testing among ovarian cancer patients in an era of histotype-specific approaches to ovarian cancer prevention

**DOI:** 10.1186/s12885-018-4153-8

**Published:** 2018-03-05

**Authors:** Gillian E. Hanley, Jessica N. McAlpine, Dianne Miller, David Huntsman, Kasmintan A. Schrader, C. Blake Gilks, Gillian Mitchell

**Affiliations:** 1 Department of Obstetrics & Gynaecology, University of British Columbia, Vancouver, BC Canada; 2 Pathology & Laboratory Medicine, University of British Columbia, Vancouver, BC Canada; 3 Department of Medical Genetics, University of British Columbia, Vancouver, BC Canada

**Keywords:** *BRCA*, Ovarian cancer, Risk reduction, Histology-based referral

## Abstract

**Background:**

Identifying female carriers of *BRCA1* and *BRCA2* mutations is imperative for prevention of ovarian cancer and breast cancer. There are five major histologic subtypes of ovarian cancer and high grade serous cancer (the most common) is reported in 75–100% of *BRCA1* and *BRCA2* mutation carriers. We examined histology-based referral to the Hereditary Cancer Program following an educational prevention campaign recommending *BRCA1* and *BRCA2* mutation screening for all high-grade serous cancer patients.

**Methods:**

We conducted a population-based retrospective study in the province of British Columbia, Canada that included all patients visiting the Hereditary Cancer Program for genetic counselling for *BRCA1* and *BRCA2* mutation between 2001 and 2014. We examined the difference in rates of *BRCA1* and *BRCA2* testing between serous cancer patients and endometrioid and clear cell cancer patients using a differences in differences analysis. We also calculated the mean number of family members tested for every *BRCA1* and *BRCA2* identified ovarian cancer patient before and after the educational campaign.

**Results:**

There were 5712 women tested for a *BRCA1* and *BRCA2* mutation at the HCP between 2001 and 2014, 887 of which had previously received a diagnosis of ovarian cancer. By 2013, 43% of serous cancer patients were being tested for *BRCA1* and *BRCA2* mutations compared with 20% of endometrioid and clear cell patients (*p* < 0.001). The mean number of family members tested for each *BRCA1* and *BRCA2* positive ovarian cancer patient increased after the educational campaign from 2.54 to 3.27 (*p* = 0.071), and the number of family members identified as BRCA positive also increased significantly.

**Conclusions:**

Recommendations for histology-based referral significantly increased the likelihood of serous cancer patients being tested for BRCA mutations. There was also an increase in the number of carrier tests performed for each *BRCA1* and *BRCA2* index ovarian cancer patient.

**Electronic supplementary material:**

The online version of this article (10.1186/s12885-018-4153-8) contains supplementary material, which is available to authorized users.

## Background

Ovarian cancer is the leading cause of death due to gynecologic malignancy and the fifth most common cause of cancer deaths in developed countries. While the general population lifetime risk of ovarian cancer is low at 1.4% [1], women at high-risk of developing the disease due to their inheritance of a germline *BRCA1* and *BRCA2* mutation have an average cumulative risk of between 40% to 75% and 11% to 34%, respectively [2–5]. Inherited germline mutations of *BRCA1* and *BRCA2* have been reported in 11% to as high as 25% of all invasive ovarian carcinomas [6–10]. Women with germline mutations in *BRCA1* and *BRCA2* are also predisposed to autosomal dominant hereditary breast cancer.

Identifying female carriers of *BRCA1* and *BRCA2* mutations is imperative for prevention of ovarian cancer and breast cancer. While screening for ovarian cancer has not been demonstrated to be of benefit [11] risk reducing interventions can be undertaken. Use of oral contraceptive pills has been shown to decrease risk of ovarian cancer in *BRCA1* and *BRCA2* mutation carriers without significantly increasing the risk of breast cancer [12] and risk-reducing bilateral salpingo-oophorectomy is highly protective reducing ovarian cancer and overall mortality by 80% and 60% respectively following surgery [13–15]. Enhanced screening for breast cancer can also be undertaken.

Histomorphologic classification of epithelial ovarian cancer, often aided by immunohistochemistry, is highly reproducible and delineates five major histologic subtypes of ovarian cancer: high-grade serous (HGSC), clear cell, endometrioid, mucinous and low-grade serous. [16] HGSC is the most common, accounting for approximately 70% of invasive ovarian carcinomas and responsible for 90% of mortality in this disease. [17, 18] HGSC is the histotype reported in 75–100% of *BRCA1* and *BRCA2* mutation carriers who develop ovarian carcinoma. [6, 8, 10, 19–21] Our previous research has illustrated that *BRCA1* and *BRCA2* germline mutations were present in over 20% of women who underwent surgical staging in British Columbia with pathology confirmed HGSC. Notably, 19% of *BRCA1* or *BRCA2* mutation carriers identified did not have a family history suggestive of hereditary breast and ovarian cancer syndrome and thus would not have been referred for, nor qualified for hereditary cancer testing [10].

Due to concerns of missed opportunities for referral, in September of 2010, our gynecologic tumor group in British Columbia initiated a province-wide ovarian cancer prevention initiative, which strongly emphasized the importance of referring HGSC patients for hereditary cancer counselling and index *BRCA1* and *BRCA2* genetic testing at BC’s coordinated site for publicly funded hereditary cancer genetic testing, the sole site of *BRCA1* and *BRCA 2* mutation testing in the province. In the early years of this recommendation the hereditary cancer program in fact accepted any non-mucinous ovarian carcinoma for referral, as histotype assignment was not as reliable. However, the principal focus was increased capture of HGSC carcinomas and in November 2011 our pathology department generated a reflex statement with their reports stating “high-grade serous carcinoma of the ovary/fallopian tube/peritoneum is associated with a *BRCA1* or *BRCA2* mutation in over 20% of patients. We recommend this patient be referred to the BCCA Hereditary Cancer Program.” The emphasis on HGSC patients resulted from the fact that studies done with central expert pathology review report that 95–100% of ovarian cancer patients with germline *BRCA1* or *BRCA2* mutations had invasive serous ovarian carcinomas, in studies where there was central pathology review using current diagnostic criteria [10, 20, 21].

Herein we present the provincial statistics on uptake of index *BRCA1* and *BRCA2* genetic testing in BC before and after the 2010 campaign comparing rates of testing between serous cancer patients and endometrioid and clear cell ovarian cancer patients. We also examine numbers of carrier tests performed for family members of each *BRCA1* and *BRCA2* mutation positive identified ovarian cancer patient, as prevention efforts rely on uptake of carrier testing in family members who have yet to be diagnosed with ovarian cancer.

## Methods

We conducted a population-based retrospective study of all women who visited the HCP between 2001 and 2014 in the Canadian province of British Columbia (population of 4.6 million) for index *BRCA1* and *BRCA2* mutation testing. This is the sole source of *BRCA* mutation testing in British Columbia, and thus is a population-based dataset for all patients tested during this study period. We were unable to capture women who were referred to the HCP but chose not to visit the HCP in our dataset. With approval of all data stewards, we used Population Data BC to access the BC Cancer Registry data for 1985 to 2013, linked with vital statistics death data [22, 23]. Thus we have one more year of data on index *BRCA1* and *BRCA2* genetic testing allowing for a lag time between diagnosis and testing and more importantly for having family members carrier tested once an index patient is identified. All inferences, opinions, and conclusions drawn are those of the authors and do not reflect the opinions or policies of the Data Stewards. Out of concern for women’s privacy and according to our agreement with the relevant data stewards we do not report cell sizes smaller than 5. In these cases, we report approximate percentages.

In BC cancer is a reportable disease and all cases are entered into the provincial Cancer Registry. The registry sources include hematology and pathology reports, death certificates, hospital reports, and cancer treatments. The data available include details about the type of cancer diagnosed and the date of diagnosis. These data were linked with data from BC’s Hereditary Cancer Program. Ethics approval was obtained from the University of British Columbia Behavioural Research Ethics Board.

We accessed data from the HCP including information on the gene tested, the results of the test, the date of the first visit to the HCP, whether the patient was an index or carrier patient, as well as data on family members who were also tested at the HCP, including the degree of relation, gender and the number of first, second, third and fourth degree relatives. We classified patients as having *BRCA1* and *BRCA2* mutation testing if they had either *BRCA1* or *BRCA2* mutation testing or both.

The data from the BC Cancer Registry was accessed for all patients who visited the HCP. We classified patients as having ovarian cancer if they were in the registry with an *International Classifications of Disease* (ICD-10-CA) code of C56.x or C48.x or a cancer site description of “Ovary” or “Fallopian tube”. We used the *International Classification of Diseases for Oncology (ICD-O-3)* morphology codes to assign histologic subtype (see Additional file 1 for details on morphology codes). The codes are unable to distinguish between high grade and low-grade serous cancers, and thus we have classified them as serous cancers. There are also ovarian cancers with morphology codes that are not detailed enough to classify into histologic subtypes and were considered “unknown” histologic subtype. These cancers were excluded from all subtype-specific analyses.

### Statistical analysis

To clearly assess the effects of the educational campaign we examined differences rates of testing between serous cancer patients and endometrioid and clear cell cancer patients. Since the educational campaign only made recommendations around referral of HGSC, we did not expect rates of testing to change among endometrioid and clear cell cancer patients. We examined the characteristics of the women who visited the HCP following a diagnosis of serous ovarian cancers compared to women with a diagnosis of either clear cell or endometrioid ovarian cancer. We used t-tests to test for statistically significant differences between the groups for continuous variables and chi-squared tests for categorical variables.

We examined the difference in rates of *BRCA1* and *BRCA2* testing between serous cancer patients and endometrioid and clear cell cancer patients and the difference in this difference before and after the educational campaign (a differences in differences analysis). We also calculated odds ratios indicating the odds of *BRCA1* and *BRCA2* testing in serous patients compared to clear cell or endometrioid patients both before and after the educational campaign. Finally we examined the number of carrier tests in families and the mean number of family members tested for every *BRCA1* and *BRCA2* identified ovarian cancer patient and serous ovarian cancer before and after the campaign and tested whether this changed significantly using two-sided t-tests. We did the same for the number of family members identified as *BRCA* positive before and after the educational campaign.

## Results

There were 5712 women tested for a *BRCA1* and *BRCA2* mutation at the HCP between 2001 and 2014. Of these women, 887 were identified as having received a diagnosis of ovarian cancer between 1985 and 2013; however most of these cancers (811, 91.5%) were diagnosed between 2001 and 2013. This represents 21.1% of all ovarian cancers diagnosed in BC during this time period (*n* = 4201).

Table 1 outlines the histologic subtype breakdown for the 887 ovarian cancer patients who visited the HCP along with the rates of *BRCA* germline mutations by histologic subtype. This is broken down before and after the educational campaign. Due to the inclusion of codes without significant detail to classify into histologic subtypes, 18.7% of ovarian cancers among women visiting the HCP were of unknown subtype. Of the women who visited the HCP 65.5% were classified as having serous cancer, 10% were classified as endometrioid. 5.2% clear cell and 0.6% mucinous (mucinous cancer was excluded from Table 1 due to privacy concerns around small cell sizes). There were significantly more serous cancer patients tested after the campaign (increasing from 59.0% to 72.5%) and significantly fewer endometrioid, clear cell and unknown histologic subtypes. The majority of *BRCA* positive patients with ovarian carcinoma had serous carcinoma (75.6%) and rate of *BRCA* positivity increased among serous cancer patients before and after the campaign (73.8% to 79.7%); however, this difference was not statistically significant. Ovarian carcinomas in mutation carriers were uncommonly reported to be of endometrioid (*N* = 5), clear cell (absolute number not reported due to privacy restrictions, approximate percentage < 5%) or mucinous (*N* = 0) histotypes (Table 1).

**Table 1:** Histologic subtype of ovarian cancer patients who visited the HCP for BRCA mutation testing before and after the educational campaign

	BRCA mutation tested (*n* = 887)			BRCA positive (*n* = 163)		
Histologic subtype, *n* (%)	Before the campaign	After the campaign	Total	Before the campaign	After the campaign	Total
Serous	270 (59.0)	311 (72.5)	581 (65.5)	76 (73.8)	48 (79.7)	124 (75.6)
Endometrioid	60 (13.1)	29 (6.8)	89 (10.0)	5 (4.9)	< 3% ^a^	< 5%^a^
Clear cell	31 (6.8)	15 (3.5)	46 (5.2)	< 4%^a^	< 3%^a^	< 5%^a^
Unknown	95 (20.7)	71 (16.6)	166 (18.7)	19 (18.5)	10 (16.4)	29 (17.7)
*p*-value			< 0.001			0.519

^a^Suppressed due to small cell sizes so approximate percentages have been reported Mucinous cancer was excluded from this table due to privacy concerns around small cell sizes

Table 2 outlines characteristics of women with serous cancer and clear cell or endometrioid cancer. Among those who were tested for *BRCA1* and *BRCA2* mutations, serous cancer patients were significantly older and diagnosed at a later year, were of older age at the time of their first visit to the HCP, and visited the HCP in a later year. There were no significant differences between index or carrier patient status, proband status or number of first, second, third or fourth degree relatives between the groups. Serous cancer patients were significantly more likely to be *BRCA1* and *BRCA2* mutation positive with 21.3% testing positive for either *BRCA1* or *BRCA2* mutations compared to 7.5% of endometrioid or clear cell patients.

**Table 2:** Characteristics of ovarian cancer patients visiting HCP for *BRCA1* or *BRCA2* testing in British Columbia by serous status, 2001–2013

	Serous ovarian cancer *N* = 581	Endometrioid or clear cell ovarian cancers *N* = 134	*p*-value
Age at diagnosis	60.0 ± 11.1	51.3 ± 11.0	< 0.001
Year of diagnosis	2007.9 ± 4.3	2005.3 ± 5.2	< 0.001
Age at time of first visit to HCP	62.3 ± 11.4	55.1 ± 11.3	< 0.001
Year of first visit to HCP	2009.6 ± 3.4	2008.4 ± 3.3	< 0.001
Index test			
Index	564 (97.1)	129 (96.3)	
Carrier	17 (2.9)	5 (3.7)	0.237
Proband status			
Yes	527 (90.7)	116 (86.6)	
No	54 (9.3)	18 (13.4)	0.151
Number of first degree relative			
0–3	423 (72.8)	97 (72.4)	
4–6	122 (21.0)	32 (23.9)	
7+	36 (6.2)	5 (3.7)	0.455
Number of second degree relatives			
0–3	257 (44.2)	58 (43.3)	
4–6	189 (32.5)	53 (40.0)	
7+	135 (23.2)	23 (17.1)	0.180
Number of third degree relatives			
0–3	303 (52.2)	62 (46.3)	
4–6	164 (28.2)	38 (28.4)	
7+	114 (19.6)	34 (25.4)	0.290
Number of fourth degree relatives			
0–3	545 (93.8)	122 (91.0)	
4–6	14 (2.4)	^a^ < 5%	
7+	22 (3.8)	^a^ < 5%	0.323
*BRCA1* or *BRCA2* positive	124 (21.3)	10 (7.5)	< 0.001

^a^Suppressed due to small cell sizes so approximate percentages have been reported

**Fig. 1:**
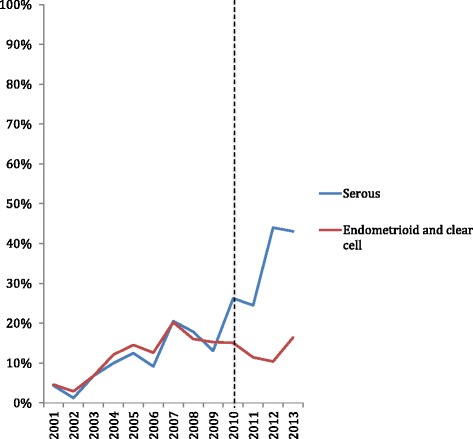
Percentage of serous cancer patients and endometrial and clear cell patients seen at HCP annually

Figure 1 outlines the percentage of all BC serous cancer patients and endometrioid or clear cell ovarian cancer patients who were tested for *BRCA1* and *BRCA2* mutation status over time. The educational campaign is marked with a dotted line. Figure 1 clearly illustrates that rates of index *BRCA1* and *BRCA2* genetic testing were similar between serous cancer and endometrioid or clear cell cancer patients before 2010. After 2010 a higher proportion of serous cancer patients were being tested for *BRCA1* and *BRCA2* mutations. By 2013, 43% of serous cancer patients were being tested for *BRCA1* and *BRCA2* mutations compared with 20% of endometrioid and clear cell patients. Differences in differences analysis reveals that the difference in testing rates between serous cancer patients and endometroid and clear cell patients before and after the campaign was significant (*p* < 0.0001). Table 3 also reports odds ratios of receiving *BRCA1* and *BRCA2* testing before and after the educational campaign with endometrioid and clear cell cancer patients acting as the reference category. Serous patients were not significantly more likely to receive testing before the September 2010 educational campaign, but nearly 5 times (OR = 4.70; 95% CI 2.89–7.62) more likely to receive *BRCA1* and *BRCA2* testing after the educational campaign.

**Table 3:** Odds ratio of likelihood of being seen at HCP before and after the educational campaign

	Odds of *BRCA1* or *BRCA2* testing
Before the educational campaign	
Endometroid or clear cell	1.00 (ref)
Serous cancer	1.27 (0.96–1.68)
After the educational campaign	
Endometrioid or clear cell	1.00 (ref)
Serous cancer	4.70 (2.89–7.62)

With respect to carrier testing, Table 4 outlines the mean number of family members tested per *BRCA1* and *BRCA2* positive ovarian cancer and serous cancer patient before and after the educational campaign. The mean number of family members tested per *BRCA* positive ovarian cancer patient and serous ovarian cancer patient increased after the campaign from 2.54 to 3.27 and from 2.60 to 3.36 respectively; however, neither increase was statistically significant at the 95% confidence level. There were significantly more family members identified for each *BRCA* positive ovarian cancer patient (1.62 vs. 2.18, *p* = 0.009) and serous ovarian cancer patient (1.64 vs. 2.30, *p* = 0.019) after the educational campaign. We were unable to test the differences for *BRCA *positive endometrioid and clear cell cancer patients because of small numbers.

**Table 4:** Average number of carrier tests per BRCA identified patients before and after the educational campaign

Index patient	Before campaign	After campaign	*p*-value
Carrier tests per BRCA positive ovarian cancer patient	2.54 (2.48)	3.27 (2.60)	0.071
Family members identified as BRCA positive	1.62 (1.10)	2.18 (1.63)	0.009
Carrier tests per BRCA positive serous cancer patient	2.60 (2.3)	3.36 (2.7)	0.098
Family members identified as BRCA positive	1.64 (1.10)	2.29 (1.75)	0.012

## Discussion

Following the 2010 educational campaign recommending that all HGSC patients be referred to the HCP for germline *BRCA1* and *BRCA2* testing, rates of testing did significantly increase compared to rates prior to the recommendation and compared to endometrioid and clear cell cancer patients. This suggests that physicians (including family practitioners, general obstetrician gynecologists and medical and gynecologic oncologists) responded to the recommendations made in 2010. This is consistent with the observed high uptake of surgical removal of fallopian tubes at the time of routine gynecologic surgery, which was also recommended in the September 2010 ovarian cancer prevention educational campaign [24]. The educational campaign was supported by the pathology department initiating a reflex comment in all pathology reports with the diagnosis of HGS fallopian tube, ovarian, and primary peritoneal carcinoma in order to prompt referral to the HCP for these women.

The finding in our manuscript that 21.3% of serous ovarian cancer patients were germline *BRCA1* or *BRCA2* mutation carriers is consistent with previous research reporting a 20% and 23% germline mutation rate in HGSC patients [9, 10, 25]. However, our findings differ in that Schrader et al. [10] previously reported that germline BRCA mutation were exclusively associated with high-grade serous histology. The Schrader series (*n* = 131) had undergone extensive pathology review with additional immunohistochemistry performed to aid in histologic subtype assignment. Cases that could not be assigned histotype with confidence were excluded. Although there have been considerable advances in categorization of epithelial ovarian cancer subtypes with high interobserver agreement in histotype assignment [26] for this disease, our large series of included cases (*n* = 887) include cancers subtyped prior to these publications, and most cases did not have the benefit of additional immunohistochemical tests to help characterize challenging cases. Thus we expect that many cases reported as clear cell, endometrioid carcinomas, or of unspecified type, from historical cohorts found to have BRCA mutations, were actually HGSC. Consistent with this Alsop et al. indicated that in their Australian cohort 8 of the 10 designated endometrioid cancer patients and 3 of 4 clear cell cancer patients identified with *BRCA1* or *BRCA2* mutations were reclassified as HGSC following immunohistopathology review, so that considerably less than 5% of patients with either endometrioid or clear cell carcinoma were found to have a germline BRCA mutation [9]. In this manuscript, we report a small number (*n* = 10, 7.5%) of germline *BRCA1* and *BRCA2* mutations found in women diagnosed with endometrioid and clear cell cancer on their initial pathology reports. The vast majority of these mutation carriers were diagnosed before 2005 prior to the changes in epithelial ovarian carcinoma histotyping. We hypothesize that many of these cancers also were high-grade serous histology.

We are encouraged by the significant increase in rates of *BRCA1* and *BRCA2* mutation testing following the educational campaign, however, it remains that only 43% of serous cancer patients were ultimately tested by the end of our study period. There is also substantial room for improvement in carrier testing family members when a mutation has been identified. While the number of family members tested for each *BRCA1* or *BRCA2* positive ovarian cancer patient increased following the educational campaign, the mean number of family members tested was 3.27 following the campaign. Given the high frequency of germline *BRCA1* and *BRCA2* mutations in women with HGSC and the high penetrance of these genes, it is imperative that every effort is made to identify women and their family members. For the woman with HGSC there are immediate predictive and prognostic implications as patients carrying germline *BRCA1* and *BRCA2* mutations have been shown to have improved rates of progression-free and overall survival and more frequently respond to platinum and non-platinum-based regimens than mutation-negative patients [9]. Germline mutation status is also increasingly relevant in guiding treatment with oral poly (ADP-ribose) polymerase inhibitors, with highly favorable response rates demonstrated, even in heavily pretreated individuals, in women with *BRCA1* or *BRCA2* mutations [27–29]. Finally, additional screening for breast cancer and risk-reducing interventions through chemoprevention and surgical procedures can be undertaken by the individual and family members who are known *BRCA1* or *BRCA2* germline mutation carriers to prevent future cancers.

The population-based nature of this study and its inclusion of all women seen at the HCP in British Columbia between 2001 and 2014 is an important strength of this study; however, the study is not without limitations. Our reliance on the ICD morphologic codes to classify tumours into histologic subtypes likely introduced some misclassification, as previously described. In addition, we were unable to examine referral patterns to the HCP to discern who was referred but did not follow up and why (patient decision vs. physician team). We expect that rates of referral were higher than the number of women visiting the HCP, but we are unable to quantify this difference. Finally, there were other events affecting the rate of *BRCA1* and *BRCA2* mutation testing during our study period, most notably Angelina Jolie’s announcement regarding her *BRCA1* status in May of 2013, which increased demand for genetic counselling considerably. However, because there was a considerable length of time between the educational campaign and Angelina Jolie’s announcement, we can conclude that rates of testing among HGSC patients increased following the educational campaign and not as a result of Angelina’s Jolie’s announcement (95% confidence level).

## Conclusions

Our research suggests that the 2010 educational campaign followed by the change in the pathology reports did increase *BRCA1* and *BRCA2* mutation testing rates among serous cancer patients in BC. There was also an increase in the number of carrier tests performed for each positive *BRCA1* and *BRCA2* ovarian cancer patient. We anticipate that the rate of identification of germline *BRCA1* and *BRCA2* mutation carriers will rapidly increase as we move towards new models of genetic testing in BC designed to accelerate the time to genetic diagnosis. We expect that testing will soon be initiated by the treating physician (near time of diagnosis) [30, 31]. Other jurisdictions are trialing methods to improve their genetic assessment rates such as the introduction of “opt-out” genetics referrals, which have dramatically improved assessment rates within a relatively short period of time [32]. Importantly, with the more rapid identification of new families, it will be critical to ensure there is translation of the preventative benefit in identifying germline mutation carriers through optimized role out of cascade carrier testing in the family. Future research should investigate whether the women identified as *BRCA1* and *BRCA2* positive through carrier testing are undergoing effective risk-reducing interventions.

## Additional file

Additional file 1: ICD-O morphology codes for histologic subtyping. (DOCX 13 kb)

## Abbreviations

HCP: Hereditary Cancer Program
HGSC: High grade serous ovarian cancer
