# Supplementary material for: A population-based analysis of germline BRCA1 and BRCA2 testing among ovarian cancer patients in an era of histotype-specific approaches to ovarian cancer prevention
Source: BMC Cancer. 2018 Mar 5;18:254. doi: 10.1186/s12885-018-4153-8 (PMC5838948; doi:10.1186/s12885-018-4153-8)
Supplement: Supplementary file 1 — ICD-O morphology codes for histologic subtyping. (DOCX 13 kb) [file 12885_2018_4153_MOESM1_ESM.docx]

Additional file 1. ICD-O morphology codes for histologic subtyping

| Histologic subtype | ICD-O morphology codes |
| --- | --- |
| Serous | 8441/X; 8442/X; 8460/X; 8461/X; 8462/X; 8463.X |
| Endometrioid | 8380.X; 8560/X; 8570/X; 8381/X |
| Clear Cell | 8310/X; 8443/X; 8444/X |
| Mucinous | 8470/2; 8470/3; 8471/3; 8480/3 |
